# A Retrospective of Project Robo Raven: Developing New Capabilities for Enhancing the Performance of Flapping Wing Aerial Vehicles

**DOI:** 10.3390/biomimetics8060485

**Published:** 2023-10-12

**Authors:** Hugh A. Bruck, Satyandra K. Gupta

**Affiliations:** 1 Department of Mechanical Engineering, University of Maryland, College Park, MD 20742, USA; bruck@umd.edu; 2 Aerospace and Mechanical Engineering Department, University of Southern California, Los Angeles, CA 90089, USA

**Keywords:** flapping wing air vehicles, bioinspired robotics, autonomous robots, multifunctional wings, independent wing control

## Abstract

Flapping Wing Air Vehicles (FWAVs) have proven to be attractive alternatives to fixed wing and rotary air vehicles at low speeds because of their bio-inspired ability to hover and maneuver. However, in the past, they have not been able to reach their full potential due to limitations in wing control and payload capacity, which also has limited endurance. Many previous FWAVs used a single actuator that couples and synchronizes motions of the wings to flap both wings, resulting in only variable rate flapping control at a constant amplitude. Independent wing control is achieved using two servo actuators that enable wing motions for FWAVs by programming positions and velocities to achieve desired wing shapes and associated aerodynamic forces. However, having two actuators integrated into the flying platform significantly increases its weight and makes it more challenging to achieve flight than a single actuator. This article presents a retrospective overview of five different designs from the “Robo Raven” family based on our previously published work. The first FWAVs utilize two servo motors to achieve independent wing control. The basic platform is capable of successfully performing dives, flips, and button hook turns, which demonstrates the potential maneuverability afforded by the independently actuated and controlled wings. Subsequent designs in the Robo Raven family were able to use multifunctional wings to harvest solar energy to overcome limitations on endurance, use on-board decision-making capabilities to perform maneuvers autonomously, and use mixed-mode propulsion to increase payload capacity by exploiting the benefits of fixed and flapping wing flight. This article elucidates how each successive version of the Robo Raven platform built upon the findings from previous generations. The Robo Raven family collectively addresses requirements related to control autonomy, energy autonomy, and maneuverability. We conclude this article by identifying new opportunities for research in avian-scale flapping wing aerial vehicles.

## 1. Introduction

Birds have long fascinated and delighted us with an impressive array of shapes, sizes, and capabilities. From the effortless soaring of the Wandering Albatross to the maneuvering precision of the Ruby-Throated Hummingbird, birds are able to move in nearly any environment by flapping their wings. As engineers and designers, we are interested in understanding how nature has generated such a versatile and effective solution to flight and, of course, how we might try to exploit flapping wing flight in engineered systems. Some of the specific features of bird wings that make them so effective for flight include:

- Wings are lightweight and aerodynamically shaped, which helps to reduce drag.
- They are flexible, which allows them to change shape during flight to generate lift and thrust.
- Wings are powered by strong muscles, which allow birds to flap their wings rapidly and generate a lot of power.

There has been a considerable amount of interest in taking inspiration from birds and building flapping wing aerial vehicles (FWAVs). Several recent survey papers provide a comprehensive review of the literature [1,2,3,4,5]. We will not attempt to provide a comprehensive review. We instead focus on highlighting major research areas with a few representative papers. The work in this field began by understanding the aerodynamics of bird flight. There has been significant work in wing design [6,7,8,9,10,11,12,13,14]. Some research focused on how to efficiently transmit rotary motion of the motors to flapping motions of wings [15,16]. Tail design and body design are also important research problems [1] Research has also explored how to design controllers for FWAVs [17,18,19,20,21,22,23,24,25,26]. There has been a focus on autonomous FWAV flights [27] and improving energy efficiency [28,29]. Recent efforts have also focused on improving maneuverability [30,31,32] and performing manipulation from FWAVs [33]. Moreover, there is a burgeoning interest in creating flapping wing platforms that take inspiration from a wide array of flying creatures. This includes exploring the flight mechanisms of bats, which can fly without tail structures [34,35,36], as well as drawing insights from flying insects for the development of miniaturized flapping wing aerial vehicles (FWAVs) capable of operating at higher flapping frequencies [37,38,39,40,41,42,43,44,45,46,47,48]. Additionally, researchers are even exploring the possibilities of designing FWAVs inspired by the flight characteristics of dinosaurs [49].

Our group started working on flapping wing flight in 2005 at the University of Maryland with a focus on the coupling of actuators with flexible wing structures to enhance flight performance. Designing and building robotic birds are challenging tasks, despite the apparent simplicity of the idea—birds flap flexible wings at the right speed to generate thrust and propel forward to move through the air and generate lift to stay afloat. The reality is that many proposed flapping wing concepts have looked feasible on paper, but failed during flight tests, primarily because of the unstructured nature of air. Unlike a land-based robot, failures for robotic birds in untethered flight lead to severe damage to the platform that can significantly slow down progress in the development of FWAVs.

**Figure 1:**
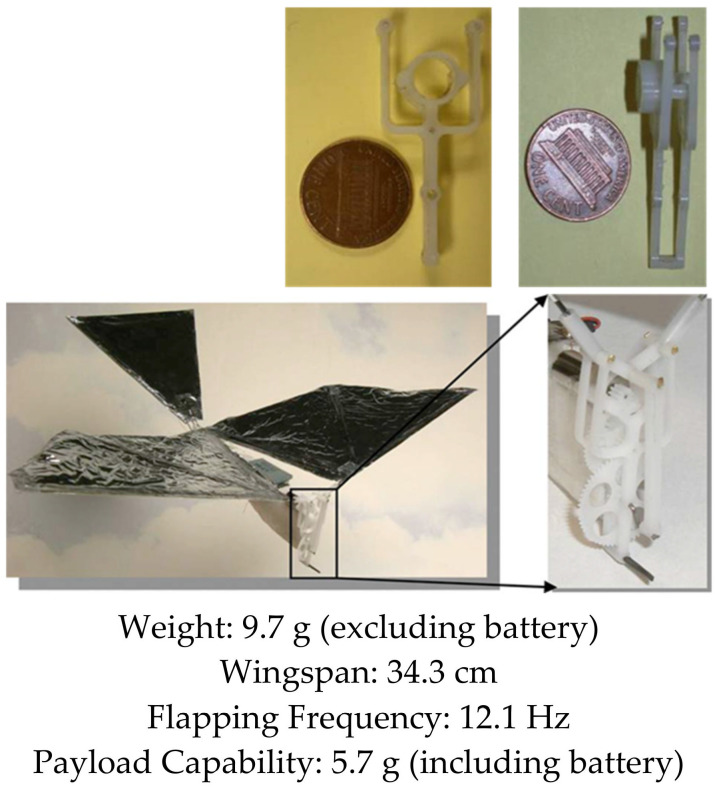
Small Bird. Flight Video: https://www.youtube.com/watch?v=RaAkboXhB9g&t=1s (accessed on 26 September 2023).

**Figure 2:**
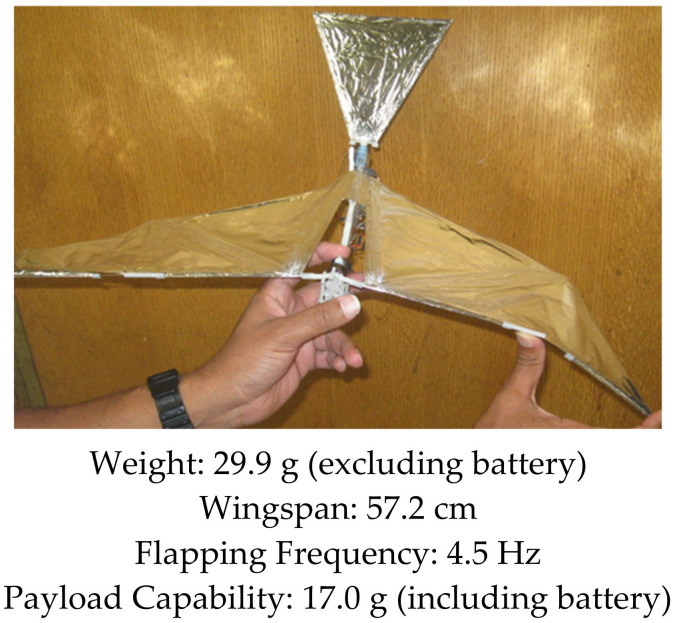
Big Bird. Flight Video: https://www.youtube.com/watch?v=q1IO3aytOLk (accessed on 26 September 2023).

**Figure 3:**
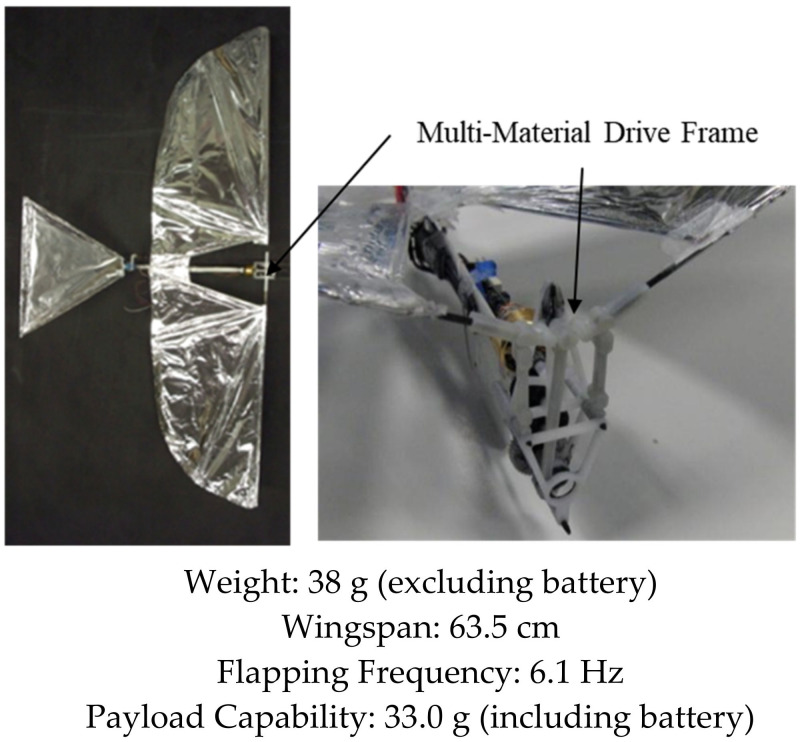
Jumbo Bird. Flight Video: https://www.youtube.com/watch?v=CtmSOUsUXfw (accessed on 26 September 2023).

Over the years, our research group has developed several bird-inspired FWAVs, both for research and as practical flying prototypes capable of transmitting live video, morphing wings, and carrying heavier payloads relative to the weight of the platform. Our first successful flight of a robotic bird was in 2007, where we created a small bird weighing less than 10 g capable of flying, climbing, and maneuvering using a single brushless DC motor to drive the wings in unison (see Figure 1). However, it was too light to fly outdoors and too delicate to carry sensors. A manufacturing process that concurrently optimizes injection mold design of drive components and their functionality was demonstrated in the Small Bird vehicle in 2008 [50]. To explore the flight performance of a variety of vehicle and wing designs, a custom testing apparatus was created to characterize force production of flapping wing vehicles [51]. These test results enabled us to design wings for a 28 g platform named “Big Bird”, which had an enhanced payload capacity enabling it to carry a camera and transmitter for live video feeds, while also improving outdoor flight dynamics by increasing wind resistance (see Figure 2). This design incorporated passive wing folding to provide decreased reliance on forward speed for lift generation through an asymmetry in wing area during the upstroke and downstroke [52]. Further enhancements in payload capacity and endurance were achieved with Jumbo Bird, which weighed 38 g, and new techniques were also developed for experimental characterization of performance (See Figure 3) [53]. As the size and power increased, we were able to generate enough lift to carry a small camera and obtain a “bird’s eye view”, while enough thrust was generated with the wings to fly in windy conditions.

While we were able to achieve a design that can fly and maneuver with a single motor, we realized that we still lacked an important capability possessed by birds: *independent wing control*. With this capability, birds are capable of performing a variety of aerobatic maneuvers, like perching, dodging obstacles, hovering, and flying in gusty winds. Unfortunately, without this maneuverability, our robot birds would crash, which necessitated redesigning the entire platform from the ground up and repeating all the flight testing (and subsequent crashing) required to refine the design. While the single motor was a good approach for efficient flight, our desire for maneuverability could not be achieved by continuing to evolve our design using this actuation mechanism. Therefore, we needed a completely different concept for powering the wings in order to realize a bird with completely independent wings that could be programmed with any arbitrary motion profiles. This led to the conception of “Robo Raven”, where the goal was to create a FWAV platform with the size scale of a raven, with each wing being powered by its own programmable servo motor, which would simulate the way that muscles power the wings of birds.

This article presents an overview of five different FWAV designs from the Robo Raven family:

- *Robo Raven I—*seminal platform capable of successfully performing dives, flips, and buttonhook turns, which demonstrates the potentially high degree of maneuverability afforded by independently actuated and controlled wings.
- *Robo Raven II*—platform optimized using a system-level simulation, which accounts for interaction among: (1) actuator, (2) battery, and (3) wing subsystem.
- *Robo Raven III*—platform that uses multifunctional wings to harvest solar energy enabling energy autonomy that overcomes limitations on endurance.
- *Robo Raven IV*—platform that utilizes on-board decision-making capabilities to control the platform in order to perform maneuvers autonomously.
- *Robo Raven V*—platform that exploits the benefits of fixed and flapping wing flight using mixed-mode propulsion to increase payload capacity through thrust-augmentation of induced drag on the flapping wings resulting in greater flight time and maneuverability.

Our previous publications focused on each individual design and how it offered a new capability. None of the previous papers explored how these different designs were related and how the capabilities exhibited by each platform can be leveraged to provide foundations for future research in this area. This retrospective review elucidates how each successive version of Robo Raven platform “evolved” based upon findings from previous generations. The Robo Raven family collectively addresses requirements related to control autonomy, energy autonomy, and maneuverability. We conclude this article by identifying new opportunities for research in avian-scale flapping wing aerial vehicles.

## 2. Robo Raven I: First Flight

### 2.1. A New Approach for Independent Wing Control

Starting with the techniques we previously developed for conventional flapping wing platforms utilizing a single electric motor, we changed the design for powering the robot bird. We wanted to explore the possibility of driving each wing directly by using a digital servo motor. In this design, each wing will be driven by its servo motor; therefore, this design would give us the ability to program the motion of each wing independently.

Achieving flight is challenging using two servo motors instead of a single actuator, but the research utility of this approach is greatly improved because of the ability to program flapping profiles that enabled us to explore arbitrary wing kinematics for useful maneuvering strategies, while also enabling us to optimize the profile for aerodynamic forces. For example, this platform was capable of a variety of unique maneuvers, such as asymmetric roll initiation, up–down flap asymmetry, gliding and soaring dynamics, gust rejection strategies, and blending of tail and wing steering modes. Hence, an important contribution expected from the development of Robo Raven I was to provide the research community with a flight-capable platform to explore interactions between compliant wings, arbitrary kinematics, and actuators to determine designs that are more efficient than single actuator-driven FWAVs. This was an important step in FWAV research because it provides the foundation for advanced maneuvers in future vehicles.

This section is based on our prior work reported in [54].

### 2.2. Design Requirements and System Development

Since our primary objective for Robo Raven was to develop a platform that could be used to ascertain the effect of changing wing kinematics and improving maneuverability outdoors, we needed a bigger platform than that of our previous robot birds in order to accommodate two servo actuators. Therefore, we identified the following design requirements based on our new design and prior experience with single actuator robot birds:

- Flap wings at least 0.20 m^2^ in size at 3.5 Hz to achieve flight outdoors.
- Flap each wing independently, but retain the ability to synchronize wing motions when needed.
- Achieve a minimum turning radius of 10 m for flying indoors and outside in terrain with tightly packed obstacles (e.g., trees).
- Remotely controlled from a distance of 500 m.
- Land unpowered from a height of 3 m without sustaining structural damage.

In addition to identifying the design requirements and system decomposition for Robo Raven, we identified the following critical components: (1) a wing drive subsystem that includes the programmable digital servos to control gait and wing sizing, (2) a compatible highly compliant wing design, and (3) a servo-driven tail for augmenting steering control and lift.

We began by testing a variety of wing motion profiles (i.e., wing angle vs. time), which included triangular and sinusoidal, to ensure that the highly compliant wings can maintain their shapes during the flap cycle to achieve the desired balance between lift and thrust. We also developed a method using a six-DOF load cell to measure aerodynamic forces generated during the flapping cycle. This enabled us to quickly evaluate different wing designs with different motion profiles to select the optimal one. These measurements were used to modify the conventional lift and thrust equations for flapping wings to account for blowback from wing deformations as follows:

(1) $$F_{D}=\left(kf\right)sin\left(\frac{\pi \left(\theta -\theta_{i}\right)}{\left(\theta_{f}-\theta_{i}\right)}\right)\;v=\left(1-\frac{D_{f}}{c\delta }\right)v_{0}$$

where *k* is an empirical factor used to change the drag force acting on the wing, *F_D_*, as a function of the flapping angle, *θ*, and frequency, *f*, and to account for the effects of wings deformations on the wing velocity, *v*; the wing velocity for a rigid wing, *v*_0_, is modified based on the drag force, *D_f_*, and wing compliance, *c*, and the displacement of the mid-chord of the wing if it were rigid, *δ*. Finally, a system-level design approach was developed for flight that consisted of coordinating the behavior of the aforementioned critical components (e.g., wing drive subsystem, highly compliant wings, and servo-driven tail). A normal flapping gait was developed for cruising flight conditions, and new maneuvering capabilities enabled by our novel independent wing control approach were investigated.

### 2.3. Summary of Results

**Figure 4:**
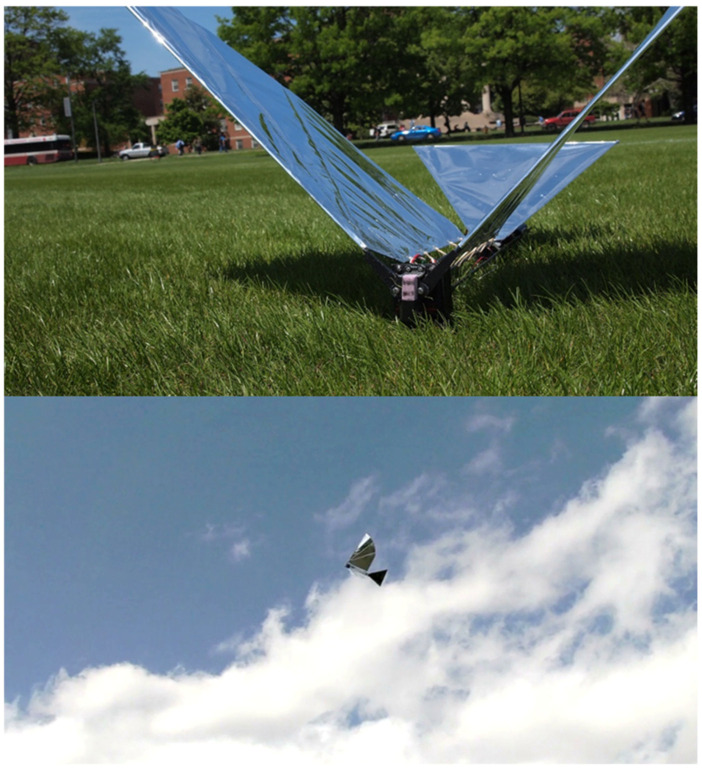
Robo Raven. Flight Video: https://www.youtube.com/watch?v=mjOWpwbnmTw (accessed on 26 September 2023).

**Figure 5:**
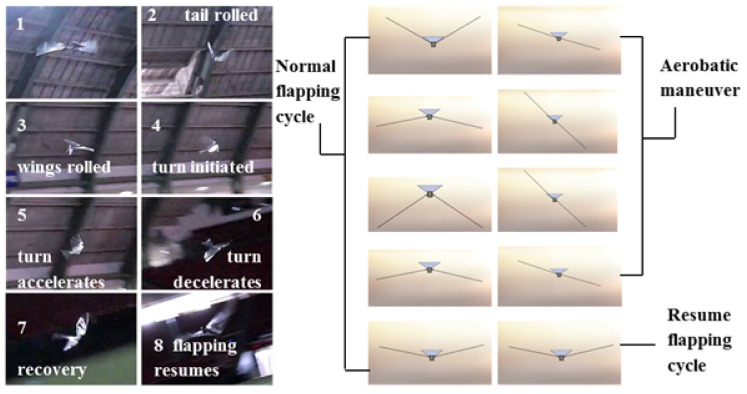
Buttonhook Turn.

We successfully built a FWAV platform that was capable of flying with independent wing control. Wing motion was controlled using programmable digital servos. Figure 4 shows the Robo Raven platform. Table 1 shows comparison of Robo Raven platform with biological ravens in terms of physical attributes. By using two programmable digital servo motors, Robo Raven was able to drive each wing independently and position them at any desired location at any desired speed. This enabled us to achieve unprecedented independent wing control of the highly compliant wing structure, which enabled us to investigate novel shape control strategies for FWAV wings by simplifying reprogramming the motors rather than having to modify the hardware. With this platform, it was possible to achieve new, more aggressive aerobatics maneuvers, which included dives, flips, steering, and buttonhook turns in an outdoor environment that is not necessarily free from wind and obstacles. Figure 5 shows Robo Raven performing a Buttonhook Turn.

As we continued to test the new vehicle, we observed some unexpected results. Sometimes, local birds like ravens, crows, and seagulls would fly nearby to investigate this strange platform in their neighborhood. As with our original design, the local birds of prey also became threatened by Robo Raven, and on more than one occasion, it would be fatally attacked by a hawk. After the initial excitement of achieving a flying platform, we quickly realized that we had to better understand the flight principles for Robo Raven in order to make further improvements. Therefore, we started working on a family of Robo Ravens, each one exploring its own interesting research questions, while retaining the common design features we discovered with the original Robo Raven.

## 3. Robo Raven II: System Modeling

### 3.1. Need for Understanding Subsystem Interactions

Robo Raven I focused solely on achieving flight using two servo motors to achieve independent wing control; but, just focusing on overcoming the obstacles involved when using two motors that prevented flight left many interesting questions still to be answered about how the system worked. This led to Robo Raven II, which focused on exploring how all the important parts of the Robo Raven design, including the motors, wings, and battery, interacted with each other. For example, a common challenge for any unmanned aerial vehicle is to increase the flight time. Naturally, a larger battery could be installed; however, it can become quite tricky to unravel the effects on performance, since the larger battery also adds weight and makes it harder to fly.

To answer the question of what happens to Robo Raven when changes like using a larger battery are made, we first built models of the three subsystems: (1) motors, (2) wings, and (3) battery. Each of these subsystems have properties affecting performance that are relatively easy to understand when viewed in isolation. However, once they are integrated into the vehicle and the components are coupled, it becomes much more interesting. As vehicle mass is increased, the loads on the wings change, which makes them deform differently. The new wing shapes lead to changes in the loads on the motors, which finally result in changing the way power is drawn from the battery. Therefore, we needed to develop a system-level modeling approach to optimize the platform design.

This section is based on our prior work reported in [55,56].

### 3.2. In-Flight Experimental Characterization Approach

Determining the effects of coupling of subsystems quickly became the focus of Robo Raven II. In order to gain insight into the problem, we had to design a suite of sensors that could be carried on-board during flight. These sensors would finally make it possible for us to measure how each of the critical system components were behaving during a flight and to use that behavior to understand and model the relationship between each component. With the help of hundreds of student hours of flight testing and processing data, we were able to develop a coupled model of the system components that described how real flights unfolded; then, we could predict how design changes would affect the flight performance.

### 3.3. Coupled Simulation Framework for System Modeling

The complexity of the powertrain for a FWAV requires simulation-based approach during design. Simulation accuracy has to be adequate to compare two different design options. The in-flight experimental characterization revealed differences between wing kinematics that were commanded and in-flight kinematics. Therefore, simulations based on idealized wing kinematics were not helpful during the design phase. We had to expand our simulation-based design approach to more explicitly consider ways that subsystems interact amongst themselves and their effect on the system-level performance. A consequence of integrating the interaction into our simulation approach was the ability to track the evolution of system-level performance of an FWAV over time, rather than providing a single estimation of performance based on a static state. In real-world applications, there will be a gradual degradation in performance associated with the drawing of battery during a mission. These performance degradations can impact the mission effectiveness and therefore need to be a part of associated design trade-off space. By providing a time history of the operational characteristics of the vehicle during each simulation run, a much more detailed depiction of how the vehicle is performing can be achieved when compared to a typical snapshot of performance that is provided in an aerodynamic code alone. There were three main tasks required to realize a coupled simulation framework for a reasonably accurate system-level model.

(1) Models for the components that account for relevant effects and can connect with each other must be selected and experimentally evaluated to provide empirical constants required to validate the predictions.
(2) Models must be connected in a way that accounts for important interactions between the drive motors, the battery, and the flexible wings to realize the required predictive capability.
(3) The approach must be demonstrated to show utility in providing design insight of the Robo Raven II platform and guidance to achieve feasible operational conditions.

**Figure 6:**
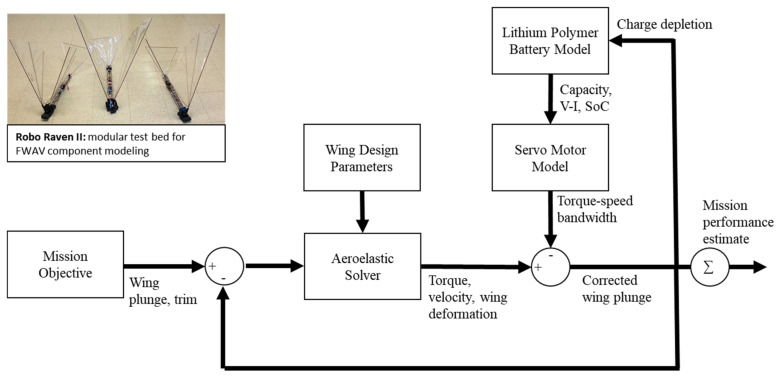
Overview of Interaction Modeling.

The simulation-based interaction modeling framework (see Figure 6) we developed provides a systematic way to compare designs. To accomplish this, we decompose the FWAV design problem into key subproblems that are each important to the system-level performance and are inherently coupled: propulsion system selection and characterization; wing design and sizing; flapping frequency and amplitude selection; and battery sizing. Using this decomposition, the most important components and subsystems influencing the system performance can be identified. Often, the selection of an appropriate actuator is the focal point because we have to often work with commercially available actuators. This initial selection sets up several important design constraints for making subsequent design decisions and is a logical starting point for the FWAV design. This selection involves a comprehensive understanding of the desired performance attributes of the vehicle, a comparison with existing natural and human-made designs, and the application of basic motor power and efficiency criteria. Flapping wing flight, natural or man-made, depends strongly on the design of the wings, including the size, shape, and flapping motions; it also depends on the design of the propulsion system used to drive the wings.

For Robo Raven II, a servo motor was chosen as the actuator, and an experimental characterization procedure was used to develop surrogate models for performance prediction. This shifted the focus towards determining how the motor characteristics and desired flapping profile affect an optimal wing size and design. Too large of a wing requires more torque than the motor can produce at a given flapping frequency and amplitude, while too small of a wing will require a flapping profile with velocities beyond the capabilities of the motor. It also means that in order to generate more torque and angular velocity, a larger motor drawing more power is required. This also requires the use of a larger battery. That, in turn, couples with the characteristics of the wing needed to generate the lift to carry the powertrain. And so, this ends up being the trade-off space for the design problem, where the focus then shifts to the wing design.

### 3.4. Wing Modeling

Flexible wings for Flapping Wing Aerial Vehicles (FWAVs) need to adapt to changing loading conditions during flight, which creates unsteady flight conditions. The performance of the drive system varies based on these conditions, making the design of flapping wing flight challenging due to the strong interdependence of subsystems. A successful wing design should effectively integrate with the propulsion system to generate enough power for lifting the vehicle while also minimizing energy consumption to enhance flight endurance and range. Designing FWAVs involves a wide range of interconnected variables, making a purely experimental approach inefficient for achieving an optimal or functional vehicle design. In the case of Robo Raven I, the focus was on developing wings for FWAVs weighing hundreds of grams. Experimental methods were employed to characterize wing performance both in controlled laboratory settings and during free flight measurements [54]. This approach generated a substantial amount of data outlining the flight envelope and subsystem parameters for acceptable operation. However, this method is time-consuming due to the extensive reliance on a large number of experimental trials to explore component interactions. Additionally, it offers limited insights into the implications of significant design changes related to key subsystems or overall system size.

Alternatively, the design problem of flapping wings can be tackled using a simulation-based approach. One of the main challenges in creating an FWAV is determining the appropriate level of modeling accuracy for generating aerodynamic predictions. Models with high fidelity offer more accurate results but come with a reduced number of deign options, and implementing real-world designs that match these complex models can be quite challenging. On the other hand, low-fidelity models offer evaluation of higher number of design options, but their predictions depend on the selected set of model inputs, which are difficult to determine without some experimental data. Given these challenges, a sensible approach involves combining an experimental method with lower fidelity simulation and modeling. This hybrid approach offers the advantages of both supporting a high number of design option evaluations and physically realistic model inputs, which are validated through testing. By doing so, we can strike a balance between accuracy and efficiency in solving the flapping wing design problem.

To overcome the limitations of experimentally characterizing the aerodynamic forces on the wings, we devised a simplified aeroelastic model. The forces generated by flapping wings in flight depend on the interplay between commanded flapping motions, power system bandwidth, and wing flexibility. Thus, creating an aeroelastic model for the wings necessitates integrating battery and actuator models to ensure a balance between the forces acting on the wings and the torque generated by the drive motor at the corresponding angular velocity. Our initial modeling efforts underscored the importance of accounting for the coupling between the motor and wings to ensure the realism of the commanded wing motions.

The aerodynamic modeling approach draws primarily from the classic strip theory method to capture aerodynamics. However, we have introduced several modifications, including unsteady terms that account for dynamic stall effects, apparent mass effects, and lift coefficient hysteresis effects. To enhance the predictions, we made critical adjustments to enforce compatibility with the drive system, incorporated more sophisticated unsteady aerodynamic predictions, and introduced a model to compute elastic wing deformations. These modifications account for differences between stiff and flexible wings, as well as between ideal and actual wing motions, and have been validated in a similarly sized FWAV under comparable flight conditions [56].

At each time step, the aerodynamic forces are calculated by solving for the normal and tangential forces based on the wing motions and deformations. The wings undergo substantial deformations and extreme angles of attack during flight, so a lift model is used to consider flow separation and post-stall behavior. The model is initialized with a commanded plunge motion and a trimmed flight condition to estimate forces and torques, which are then adjusted through a comparison to a drive system model incorporating two key effects. The first effect involves a motor model with available motor bandwidth in terms of torque and speed, while the second is a battery model that considers derating effects due to the state of charge (SOC) and high discharge rates, propagating into the motor model to update the available bandwidth. This inner loop is computed at each time step until convergence is achieved, after which the estimated charge depletion updates the battery model for the next time step. This process continues until the battery is sufficiently depleted to terminate the flight, based on the reduction in available motor bandwidth. Further details of the drive system modeling will be discussed in subsequent sections.

### 3.5. Drive System Modeling

**Figure 7:**
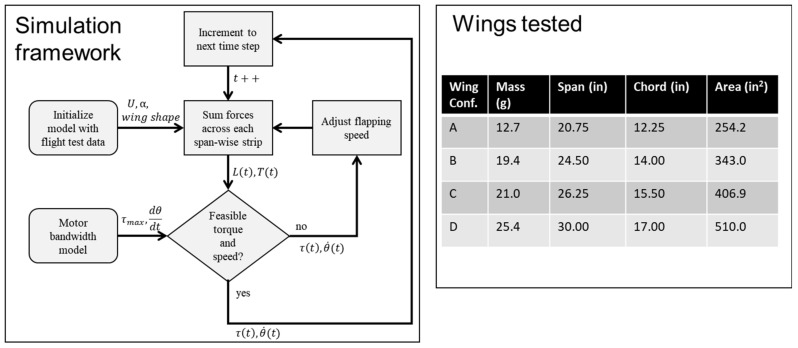
Overview of Coupled Wing–Motor Modeling Approach.

The choice of motor for the drive system plays a crucial role in analyzing flapping flight, as it determines the achievable motor bandwidth due to the available torque being contingent on rotational velocity and voltage conditions. Modeling the motor encompasses an operational range, spanning from a free-run condition with no load to a stalled motor condition at peak torque, covering all feasible battery voltage levels. For Robo Raven II, a motor model was selected based on its successful implementation in various endeavors, demonstrating highly accurate performance representation through experimental validation. Alternatively, it might be reasonable to approximate motor performance using manufacturer-published data, which includes maximum torque and maximum rotational velocity at typical reference voltages like 5.0 V and 7.4 V. Figure 7 provides an overview of the integrated wing–motor modeling approach, allowing us to capture the interactions between wing motion and motor loading. This coupling is instrumental in comprehending the dynamic relationship between the wings and the motor, which is crucial for accurate analysis.

An essential component in a comprehensive system model of flapping wing flight is the lithium polymer battery used to supply power. The battery selection must provide maximum system endurance while meeting other constraints since the available motor bandwidth experiences a significant shift during the discharge cycle. In real-world missions, the flights are more likely to terminate due to a loss of motor bandwidth at an intermediate state of charge (SOC), rather than full battery discharge. To accurately assess how a chosen battery impacts system performance, we adopted an experimentally validated battery model that encompasses all relevant aspects of battery health, environmental factors, and usage conditions. This includes runtime, multiple timescale current–voltage characteristics, and derating effects associated with rapid discharge.

The modeling approach relies on experimental characterization to set appropriate values for empirical constants. This step is necessary due to variations between battery chemistries, manufacturers, and other conditions that must be considered to maintain sufficient predictive accuracy. During the simulation process, the SOC value is updated using the Coulomb counting approach. Subsequently, the current–voltage behavior during battery discharge is modeled, allowing for variable battery capacity. The model captures the slow timescale effects associated with the first term representing open-circuit voltage, as well as the fast timescale effects, described by using a nonlinear discharge rate term and a battery plant model with exponential decay. These fast timescale effects are crucial due to the high discharge rates and rapidly changing cyclic loading conditions experienced by the Robo Raven platform.

To ensure the model accurately represents the specific make and model of batteries chosen for our application, model tuning is performed. This process involves generating a data set that captures a complete discharge process using the test stand, which then serves as input for the battery model. It is important to mention that the thermal behavior of the battery and motors have not been included in the drive system modeling. This omission is intentional, aiming to reduce the complexity of the analysis.

### 3.6. Summary of Results

**Figure 8:**
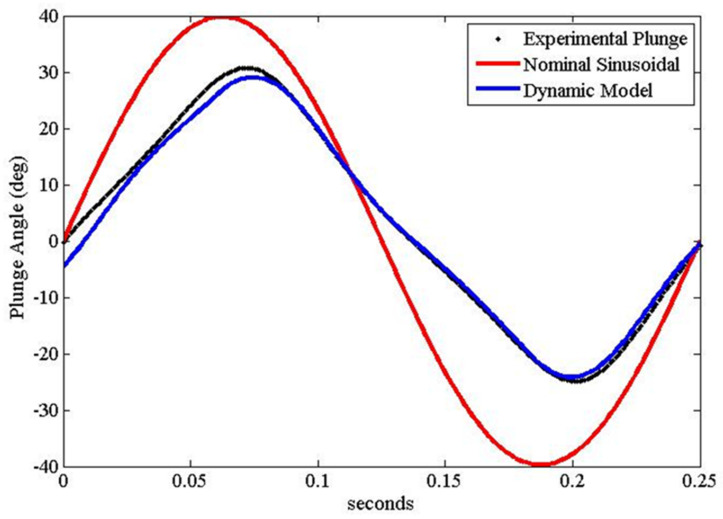
Improved Plunge Angle Prediction (based on Wing (**C**) in Figure 7).

**Figure 9:**
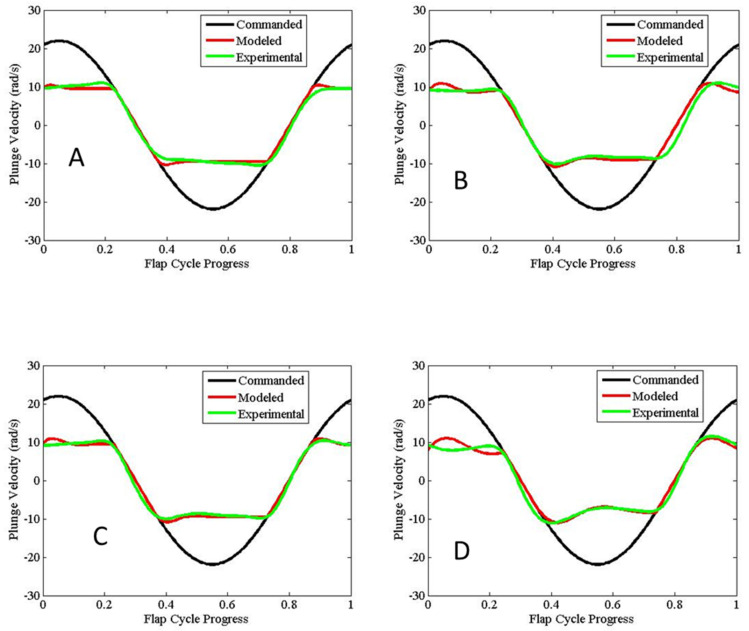
Comparison of Flap Velocity Across Wings (Letters (**A**–**D**) refer to wing types).

**Figure 10:**
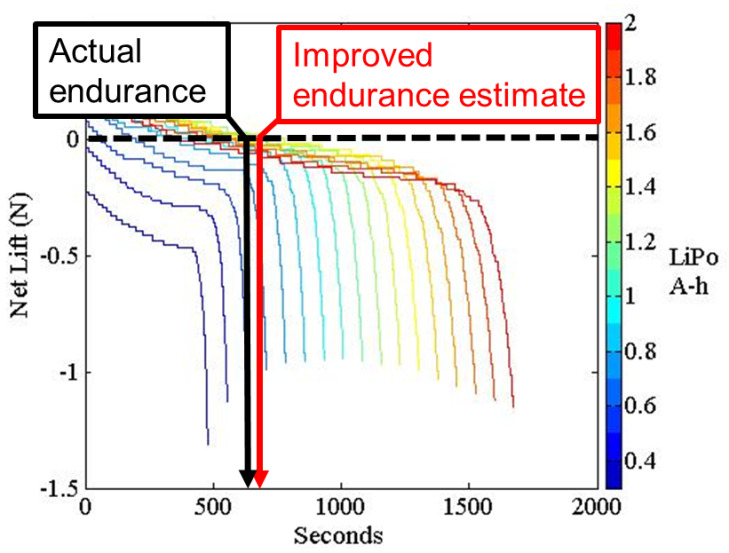
Improved Endurance Prediction Using Coupled Component Modeling.

**Figure 11:**
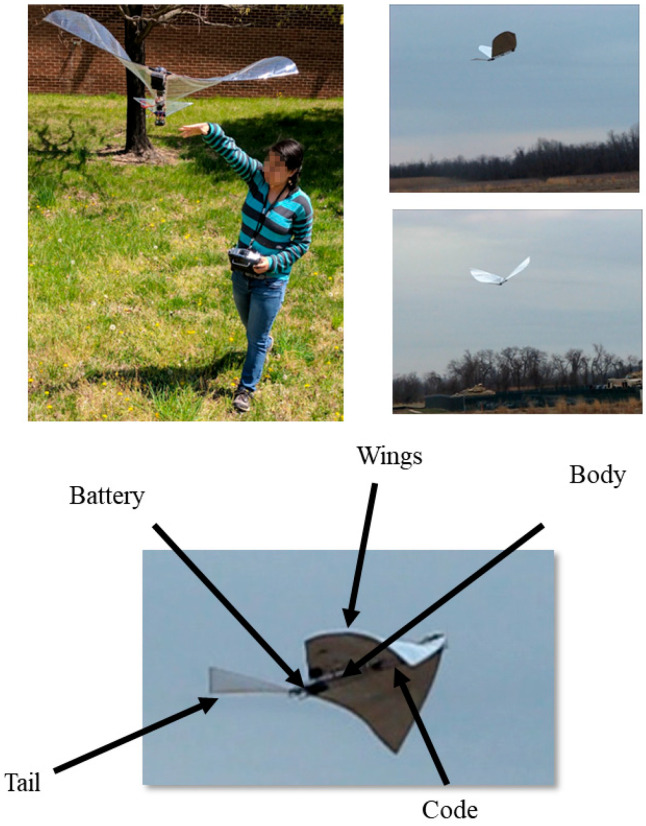
Robo Raven II: Improved system design for optimized flight performance. Flight Video: https://www.youtube.com/watch?v=q6ga9hxm6FY (accessed on 26 September 2023).

Robn Raven II demonstrated that it was possible to use a system-level simulation approach to optimize the selection of components for FWAVs. The simulation approach couples models of the (1) actuator, (2) battery, and (3) wing subsystems, which capture important effects for improving system-level performance. Figure 8 shows that our approach leads to an improved wing plunge angle prediction. Figure 9 shows that our approach leads to significant improvements in flapping velocity for different types of wings. Data presented in Table 2 show that the more accurate kinematics predicted by the coupled model reduce errors in the prediction of lift, torque, and power consumption. In order to measure torque, data from the voltage sensor, current sensor, and optical encoder were combined with the motor model. Figure 10 shows that using a coupled component modeling approach leads to improved endurance prediction. Figure 11 shows the Robo Raven II platform that was designed and fabricated for the optimized flight performance.

There are many advantages to adopting simulation-based design approach for designing FWAVs:

- Predicted flight endurance and lift production will reflect interactions under real-world conditions leading to more optimal designs.
- They can be used to make early design decisions when a lack of experimental data makes it more challenging to identify the initial specification of a feasible vehicle.
- Through subsystem modeling, it is possible to narrow down the design trade space by collecting data on just the individual subsystems, and then coupling them in the simulation.
- As a vehicle design is realized, the simulation can then be focused on a particular aerodynamic and structural regime by tuning parameters to enhance predictive accuracy and resolution. This approach ensures suitable predictive accuracy via the tunable parameters, while minimizing the amount of laborious experimental analysis that is needed.

The results of research into Robo Raven II led to some useful design changes, depending on what kind of mission you want to fly. For example, slightly larger wings can be used to improve lift and subsequent payload capacity. If you prefer to fly for a longer time, you will actually need to use smaller wings, which are more efficient. This principle holds true for birds as well. A notable example is the bar-tailed godwit, which holds the record for the longest non-stop flight. Despite having a smaller wingspan of 70–80 cm, the bar-tailed godwit outperforms birds of similar weight, such as rooks.

## 4. Robo Raven III: Solar-Powered Wings

### 4.1. Need for Energy Autonomy

One of the greatest challenges for FWAVs is overcoming their limited flight time due to the constraints of their payload. Therefore, achieving long flight times, which is critical for unmanned air systems and autonomous applications, for an FWAV using just batteries is not possible. This means there is a need to achieve energy autonomy if FWAVs are to be useful for most real-world applications. Thus, integration of energy harvesting technologies in components, such as wings, has the potential to provide this energy autonomy. This concept led to the development of Robo Raven III, which exploits the latest advances in solar cells to enhance flight performance. By integrating these components, the wing becomes multifunctional, which mirrors the design of flying animals that rarely have any components in their biological system that serve only a single purpose. Part of the key to unlocking the impressive performance of animals is mimicking this design principle, where each part provides multiple benefits. Therefore, we wanted to significantly expand the capability of FWAVs by using multifunctional wings.

This section is based on work we previously reported in [57,58,59].

### 4.2. Design Framework for Multifunctional Wings with Solar Cells

A framework was developed for designing flapping wing aerial vehicles using multifunctional wings that consisted of

- modeling solar energy harvesting while flying;
- determining the number of solar cells based on power requirements for flight;
- determining appropriate locations for the desired number of solar cells.

A system model for flapping flight was also developed to predict payload capacity for carrying batteries to provide energy only for power spikes and to enable time-to-land safely in an area where batteries can recharge when the sun sets. Results from these models could be used to predict the flight time, *T*′, as follows:

*T*′ = *k_b_*(*k*_1_*k*_2_*F*_1_/*g* − *M_m_* − *M_s_*)/(*P* − *k_s_M_s_*) (2)

where *k_b_*, *k*_1_, and *k*_2_ are coefficients for the battery energy density, the relative wing stiffness due to integrating the solar cells, and subsequent relative change in aerodynamic lift, respectively; *F*_l_ is the lift force, *g* is the gravitational constant, *M_m_* is the mass of the platform, *M_s_* is the mass of the solar cells, *k_s_* is the power density of the solar cells, and *M_s_* is their mass.

We applied this framework to a case study for Robo Raven III, where it was (1) assumed that flexible, high-efficiency (>24%) gallium arsenide (GaAs) solar cells were used, and (2) that a powertrain with 81% efficiency could potentially replace the less efficient servos we were using. Key findings included:

- The fraction of solar flux incident on the wings during flapping was 0.63 at the lowest solar altitude.
- Using a 1.25 safety factor, the minimum value of incident solar flux for the purposes of design would therefore be 0.51.
- Aerodynamic modeling and characterization of the platform determined that integrating solar cells in the wings resulted in a loss of thrust and greater drag, but the resulting payload capacity was unaffected because of a higher lift coefficient obtained from the stiffening of the wings by the solar cells.
- A time-to-land of 2500 s was predicted, and the flight capability of the platform was validated in a netted test facility.
- A powertrain with 81% efficiency would enable the platform to fly using batteries only to provide energy for power spikes, as well as additional time to achieve a safe landing when the sun sets.

Given a system weight, actuator restrictions, and the current understanding of how to design for the aerodynamics of flapping wing flight, it can be challenging to identify a functional wing design using this framework. While we were able to successfully identify airflow characteristics over the wings that were needed to enhance flight endurance using solar cells integrated into wings, achieving these characteristics is less straightforward. Typically, a functional wing design is achieved through a time and effort intensive trial-and-error process. Consequently, when a successful wing is identified, it is often favorable to make small variations in order to maintain performance. Therefore, integrating solar cells that are thicker and less flexible than the mylar it replaces made it necessary to consider the impact of their greater stiffness on wing deformations, as well as where their in-plane wing locations were. In addition to the increased stiffness, the solar cells also weigh more than the mylar, which affects weight contribution of the wings to the total mass and increases inertial loading that affects the aerodynamic force generation when flapping due to torque limitations of the servos.

As with any uncertainty associated with implementing major design revisions or new designs, detailed analytical formulations may not allow designers to make intuitive design decisions. However, knowing which factors to tune many not always be clear given modeling complexity, while the inputs for such formulations may not be available without additional experimental characterization, which is time and resource demanding. Additionally, computational overhead for independent system models becomes a challenge for control when complex models are used. In contrast, simplified models can be more useful for initial design decisions and controller designs, but at the expense of predictive accuracy. Therefore, in order to compensate for the lack of accuracy, worst-case analysis and safety factors were used to ensure flight capabilities. Ultimately, it may be necessary to use more complex models to provide superior accuracy, assuming that the underlying assumptions are valid, while the simplified model provides design alacrity by enabling design decisions to be made in the face of otherwise overwhelming uncertainty. Using both approaches can also ensure the platform has the adequate power to prevent a loss of control that risks harm to both the vehicle and anything below it.

### 4.3. Power Management Using Solar Cells

Unlike alternative power supplies, FWAVs that rely on harvesting energy for flight must be designed to tolerate power fluctuations due to changes in the amount of incident solar flux received or due to surges in the electrical system. Their flight path will also affect power generation depending on the heading and altitude changes relative to the sun to achieve a desired flight path. For example, when a high pitch may be required to climb to altitude or a roll maneuver is executed in order to turn, the energy being harvested can change dramatically because the solar cells move relative to the body while the body itself changes relative to the incident solar flux. Additionally, the power consumption needed to maintain flight varies with changes in the angle of attack and flapping frequency associated with these maneuvers, as well as from changes in the wing area and mass due to installation of solar cells. Determining the number of solar cells required to provide system power will also be of utmost importance, which means that all of the factors we have discussed need to be taken into account when integrating solar cells into the wings. Therefore, we took the following approach to design FWAV wings with integrated solar cells:

- Solar energy harvesting was modeled to predict energy generation considering various attitudes achievable by a flapping wing aerial vehicle, as well as the effects of flapping on orientation relative to the incident solar flux.
- The number of solar cells required to meet operational power requirements was determined by considering candidate solar cell technologies and accounting for sensitivity to the orientation of the solar cells relative to the solar flux.
- To accommodate the number of solar cells needed to meet power requirements, appropriate locations of solar cells in the wings, as well as the body, were identified.
- Depending on the location, time of day, and time of year, the solar flux incident on the solar cells will also vary due to the altitude and azimuth angles.

Previous findings affirmed that power generation via solar cells varies with the angle of incidence via experimental testing. So, to implement this approach, a generic case was considered assuming a cosine relationship.

### 4.4. Summary of Results

**Figure 12:**
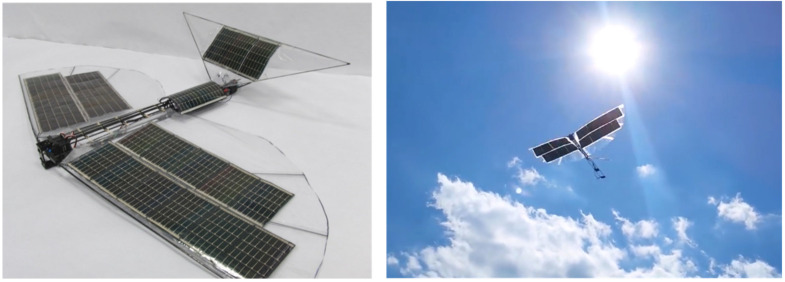
Robo Raven III: Solar-powered flapping wing flight using flexible solar cells integrated into wings. Flight Video: https://www.youtube.com/watch?v=t1_mPe8Y0V4 (accessed on 26 September 2023).

Figure 12 shows the Robo Raven III platform designed by integrating solar cells into wings. Using the modeling framework, we were able to demonstrate the following:

- an enhanced model for energy harvesting with flapping multifunctional wings that accounts for orientation of the wings relative to the sun.
- the effects of integrating HE solar cells on the payload capacity of the platform, which affects the time-to-land and power draw.
- the conditions needed to design multifunctional wings with HE solar cells to match the potential power requirements of the platform.
- A key finding was the fraction of solar flux incident on the FWAV will be only 0.94 during flight when the sun is at its zenith, and the fraction of solar flux incident on the solar cells in the wings further reduces to 0.88 due to flapping. These values reduce to 0.68 and 0.63, respectively, at the lowest solar altitude, and if a safety factor of 1.25 is employed, then the lowest value for the purposes of design will be 0.51.
- To meet power requirements, flexible HE solar cells were not only integrated into the wings, but also into the body and tail of Robo Raven III.
- A replacement motor and gear train combination with an efficiency of 81% would be capable of matching the power output of the HE solar cell to achieve flight without batteries.
- The resulting payload capacity for the battery backup was determined for flight without batteries.
- Integration of solar cells reduced thrust because of the wings being 50% stiffer with a 60% increase in drag during flapping. However, the lift coefficient of wings determined from lift force measurements without HE solar cells was determined to be 70% less than those with HE solar cells.
- Flight capability of the platform was validated through outdoor flight in a netted test facility with a backup battery.
- Electrical performance validation using test stand measurements indicated that HE solar cells improved battery discharge time by 247% to a cutoff voltage of 7.4 V for a single servo flapping the wing at 4 Hz.

By installing flexible cells into the wings, body, and tail, three improvements were made. First, the battery life was extended by providing in-flight charging from the sun’s energy. Second, and less obvious, the wing properties were altered by the solar cells, leading to improvements in the aerodynamics thanks to favorable stiffness changes. Third, we found that the output of the solar cells was also sensitive to thrust forces; therefore, these can also be used as sensors. Robo Raven III was a crucial step towards achieving a robot bird that has the potential to remain aloft for a long period of time. An exciting future vision will be a vehicle that can autonomously perch, charge up its batteries in the sun, then continue flight, all while exploiting updrafts and thermals to reduce energy consumption. It is no coincidence that this vision is so closely following the example provided by nature.

## 5. Robo Raven IV: Autonomous Flight

### 5.1. Need for Autonomous Operation

Previous generations of Robo Raven needed to be teleoperated by a human operator. The wide variety of possible flight behaviors for a flapping wing air vehicle makes this an essential piece of the overall research objective. At any time during flight, it is possible to alter the flapping range, the flapping rate, the tail angles, the body orientation, and a number of other parameters. Controlling all of this from the ground with a remote controller turns out to be a significant challenge. Therefore, FWAVs need to fly autonomously. The next step in the evolution of Robo Raven was to focus on increasing the capabilities of the platform by incorporating several autonomous behaviors.

This section is based on our prior work reported in [60,61,62].

### 5.2. Integration of Simulation and Sensing

To achieve autonomy, an autopilot with a variety of sensors on-board that measure position, velocity, and orientation during each flight was integrated into the FWAV platform, giving rise to Robo Raven IV. Using the information from its sensors, the autopilot made Robo Raven IV significantly easier to control. For example, a GPS coordinate could be uploaded, and the vehicle would go to that position and wait for more commands. More dynamic maneuvers were also developed. Given a target on the ground that the pilot wants to record on a camera, the Robo Raven IV can be commanded to dive toward the target for a perfectly smooth video. This is a very important capability for robot birds, because normally the video would be heaving up and down with each wingbeat, which makes things quite difficult to see.

The autonomy research that went into Robo Raven IV complemented our efforts with Robo Raven III in developing multifunctional wings and with Robo Raven II to model component interactions at the system-level. By combining an understanding of how the vehicle behavior will impact the performance through an on-board model-based controller, it becomes possible to enhance flight performance in ways that a ground-based pilot could never achieve. For example, in real-time, the autonomous controller may measure how the vehicle is behaving, then make adjustments to each wingbeat based on models of how the wings work and some mission objective, like maximizing range. In this way, the autonomous controller may be thought of as the brain and nervous system, controlling the way the bird behaves based on some higher-level goals for the flight.

### 5.3. Summary of Results

**Figure 13:**
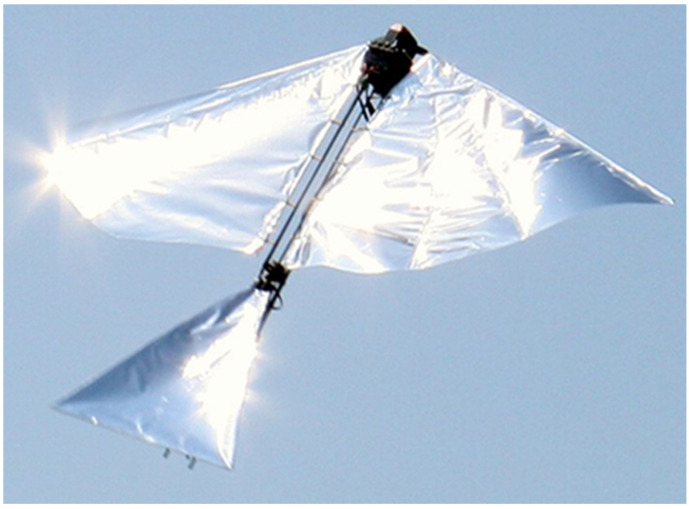
Robo Raven IV: Autonomous flight using on-board instrumentation suite to guide wing and tail adjustments. Flight Video: https://www.youtube.com/watch?v=nZ0sOFI5suw (accessed on 26 September 2023).

Figure 13 shows the Robo Raven IV platform that is capable of autonomous flight using an on-board instrumentation suite to guide wing and tail adjustments. For this demonstration, a maneuver was chosen because it was the simplest for exploring the motion control capabilities and, therefore, serves as a foundation for designing more complex maneuvers. There are several challenges with programming this maneuver:

- Variability in wind conditions makes prediction of aerodynamic forces challenging.
- Limits on payload carrying capacity restricts power due to small battery size, which subsequently limits on-board computing and sensing capabilities.
- Dive maneuvers can be designed using sophisticated control algorithms and sensors to execute highly accurate dives; they can also be designed for minimal on-board computing and sensing on-board with lower positional accuracy.

Ultimately, it may be optimal to use a combination of complex and simple control algorithms, depending on wind conditions and payload limitations. However, for this investigation, we chose to develop dive maneuvers using simple control algorithms because of the minimal on-board computing due to payload limitations of Robo Raven.

While we chose to use a simple control algorithm, it did enable us to explore two fundamental questions: (1) What is the simplest possible computational model that can provide a dive accuracy of 5 m in open loop operation mode during outdoor flight under wind speed of 3 m/s? and (2) Can the platform independently execute roll control through tail positioning and dive control through wing positioning to produce safe dive behaviors? The first question addressed dive accuracy requirements, which were set based on keeping the object of interest in the camera field-of-view despite position inaccuracy introduced by dive. Maximum wind speed was set to 30% of the vehicle’s forward velocity at a non-wind condition to ensure that there was sufficient thrust to maintain control given the positional inaccuracies. For the second question, we developed a simplified dynamics model where the wing and tail were modeled as articulated flat plates to approximately predict potential dive paths.

**Figure 14:**
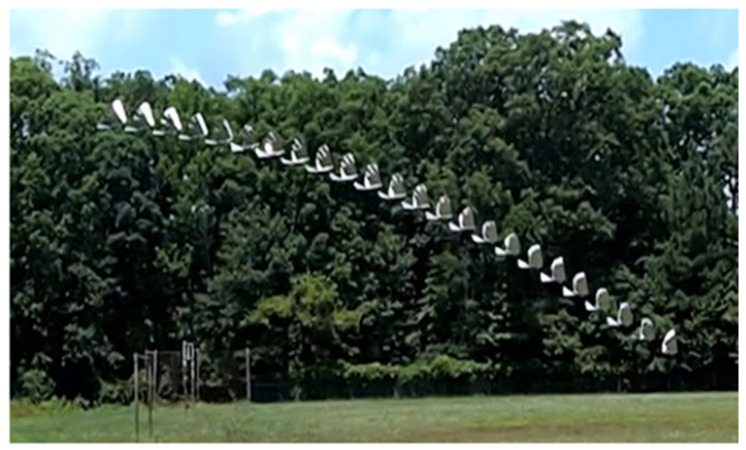
Dive in action.

**Figure 15:**
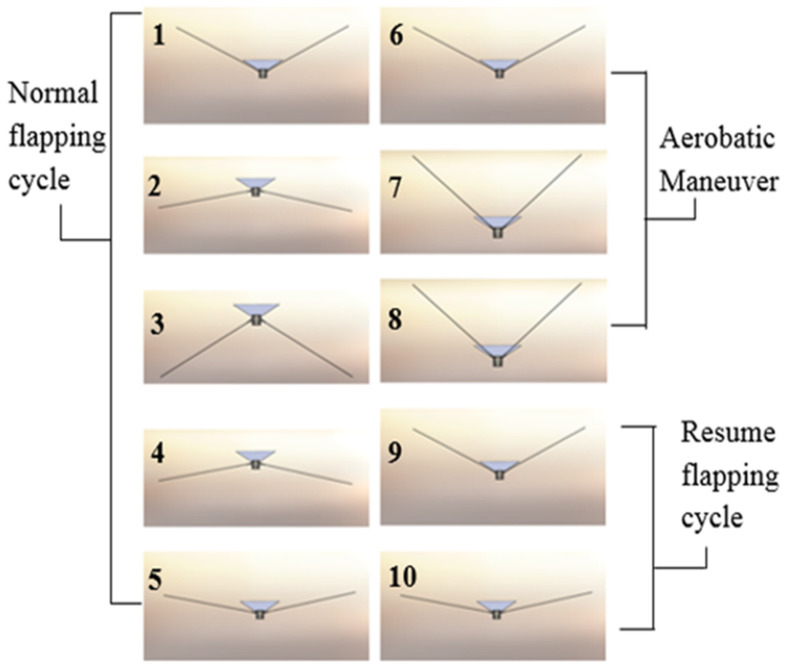
Dive maneuver simulated with CAD model.

For the plate model, the aerodynamic forces on a plate *I*, $\vec{F}$*_plate_*_(*i*)_, are determined as follows:

(3) $${\vec{F}}_{plate(i)}=\frac{1}{2}\rho_{air}C_{D}A_{plate\left(i\right)}{\left({\vec{V}}_{\left(i\right)}\cdot {\vec{n}}_{plate\left(i\right)}\right)}^{2}{\vec{n}}_{plate(i)}$$

where $\vec{n}$*_plate_*_(*i*)_ is the normal vector to the plate depending on the flapping angle and orientation of the bird, $\vec{V}$_(*i*)_ is the velocity near the center of the plate, *C_D_* is the coefficient of drag, *ρ_air_* is the density of air, and *A_plate_* is the area of the plate. While the tail of the FWAV could be modeled with a single plate because it was designed to be more rigid, the flexible wings required using five articulated plates in order to capture the effects of deformation due to the aerodynamic forces. The sum of forces on all of the plates can then be used to calculate the subsequent translational accelerations of the bird, while the associated sum of moments about the center of mass can be used to calculate the angular accelerations about the center of mass. Using this model, an algorithm was developed to choose between potential dive paths based on a desired inspection area. This enables an open loop control approach for autonomous dive maneuvers that is demonstrated on the Robo Raven platform. Figure 14 shows a series of images showing dive in action. Figure 15 shows an illustration of dive maneuver simulated using the CAD model.

Using Robo Raven IV, we were able to demonstrate that a simple flat plate model provides an accuracy of 5 m in open loop mode in outdoor flight under calm wind conditions, which is suitable for performing dive maneuvers. This model was implemented as a simple lookup table that enabled the ArduPilot to determine the desired location for executing actively controlled autonomous dives to reach a specified target location. Through the use of lookup tables, the required computational power was found to be negligible compared to the power consumed by the main servo motors. Thus, Robo Raven IV demonstrated that autonomous dives with pullouts could be executed successfully and reached within 6 m of the goal location, which is very close to the model error. As expected, pitch had little effect on the success of the dive maneuver. Independently executing roll control through tail and dive control was also found to be very reliable, since it did not lead to any failures due to excessive rolling, even after more than 100 dives were executed.

## 6. Robo Raven V: Mixed-Mode Propulsion

### 6.1. Need for Augmenting Thrust

In addition to the need for energy autonomy, there has also been a need to increase the payload capacity for Robo Raven to enable it to lift heavier loads. By increasing payload, more sensors can be added for more complex autonomous control, or more power sources can be added to increase flight time. However, achieving greater payload capacity by enhancing flapping propulsion is quite challenging due to the complexity of the design space. For example, larger wings may generate forces that exceed the limits of the actuators for the wings, leading to smaller flapping angles and diminished performance. While nature evolved birds to generate thrust by flapping wings, it was not able to evolve a structure that could do so by rotating, like propellers and rotors used for fixed wings and rotorcraft. Flapping wings and traditional propulsion used with fixed wing aircraft need not be mutually exclusive. Given the thrust provided per unit mass when using propellers (even at scale), it was postulated that a propeller-assisted flight mode might generate more thrust and, in turn, aerodynamic lift. Therefore, departing from a strictly bio-inspired approach to generating thrust has the potential of reaping benefits from multiple propulsion modes. We wanted to explore if incorporating a pair of small propellers with brushless motor drives in the rear of the bird could significantly augment thrust and the subsequent lift and payload capacity, while still providing all the benefits of flexible wings that are independently controlled. This led to the development of Robo Raven V, which was the first FWAV capable of flight using mixed-mode propulsion.

This section is based on our prior work reported in [63].

### 6.2. Propeller–Wing Interactions

Much of the research for Robo Raven V involved understanding how the propeller and flapping wing systems interacted with each other. This required designing new experiments and models, since it no longer resemble the mechanisms in nature. Through a combination of isolated tests in a laboratory and flight tests with on-board sensors, data were collected that described how the vehicle would operate using mixed-mode propulsion. Based on these data, we were able to develop models for Robo Raven V describing the benefits of mixed-mode propulsion; we could thus finally build a bird with strong flight performance capable of substantially more aggressive and dynamic maneuvers than any of the previous generations of Robo Raven.

Designing this platform posed two major challenges: First, interactions between the flapping wing and propeller-assisted flight, particularly the placement of the propellers relative to the flapping wings, need to be understood to determine if there are effects that adversely limit the aerodynamic forces that are generated. Second, adding propellers to an existing platform increases platform weight and requires additional power from heavier energy sources for comparable flight time. To gain insight into these effects, we first experimentally characterized the forces generated by each mode of propulsion independently; then, we characterized the forces while the two modes were operating together. This enabled each mode to be modeled and insight into the interactions between the two flight modes to be obtained. From our experimental investigations, we were able to determine three major findings with regards to these challenges. First, locating the propellers behind the flapping wings (i.e., in the wake) exhibited minimal coupling, as long as the propeller placement was at or below the platform centerline. Second, the additional thrust generated by the platform does increase aerodynamic lift despite the flexible wings not having a conventional airfoil design. Third, the increase in aerodynamic lift offsets the higher weight of the platform, resulting in significant improvements in payload capacity. The effect of varying operational payload and flight time for different mixed-mode operating conditions was predicted, and the trade-off between the operational payload and operating conditions for mixed-mode propulsion was characterized. Flight tests revealed the improved agility of the platform when used with static placement of the wings for various aerobatic maneuvers, such as gliding, diving, or loops.

### 6.3. Propeller Placement

**Figure 16:**
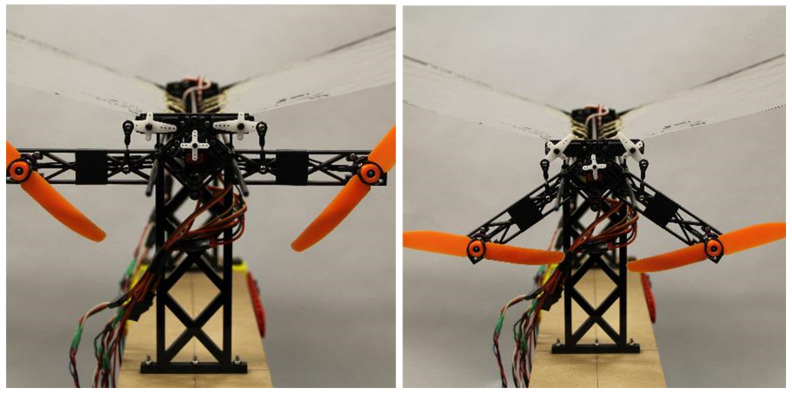
Robo Raven V Propeller Assemblies.

Potential configurations for propeller placement include (a) in front of the wings or behind the wings, and (b) above, equal with, or below the body centerline. Since Robo Raven already uses heavy servos that bias the center of mass to the front of the vehicle, it was decided to place the propellers behind the wings. It is possible to offset the bias from placing the propellers at the front of the platform by extending the length of the body and attaching the battery and controller, similar to how we were able to adjust the center of mass for the original Robo Raven platform; however, there would still be a challenge designing a mechanism to securely attach the propeller assemblies without affecting the flapping mechanism’s electrical access to the servo motors. It is also unclear how a localized change in air velocity before the wings would affect both the wind deformation and the airflow phenomena, particularly leading edge vortices (LEVs). In addition to determining the best axial location for the propellers, we also designed a mounting assembly that enabled us to investigate the effects of locating the propellers above, equal with, and below the body centerline on the coupling with the wake of the wings. (Figure 16). The propeller mounting assembly had to be longer than the propeller radius with enough stiffness to prevent deformations from the torque created by the propellers, while the distance between the back of the wing and the propeller was minimized to reduce the form factor.

Placing the propellers above the centerline pitched the nose downward because of the net moment generated by the thrust of the propellers at a distance away from the centerline. With the propellers equidistant from the centerline there will be no net moment. However, they were located directly behind the flapping wings, where any potential aerodynamic coupling would be greatest. Therefore, placing the propellers below the centerline was determined to be favorable, because the net moment generated by the thrust of the propellers counteracts the downward pitching moment from the forward mounted flapping motors; it also minimizes coupling by not being located directly behind the mid-stroke of the wings where the greatest thrust force is generated during flapping. Therefore, the optimal position of the propellers was found to be behind the wings and below the centerline.

### 6.4. Summary of Results

**Figure 17:**
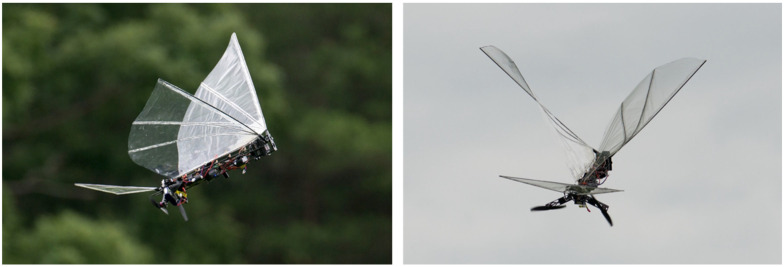
Robo Raven V Flight. Flight Video: https://www.youtube.com/watch?v=Yryz8PSAwmA (accessed on 26 September 2023). Launch Video: https://www.youtube.com/watch?v=V8FS_58_wC4 (accessed on 26 September 2023).

Figure 17 shows Robo Raven V was capable of achieving flight. Test results indicated that there is no notable cross-linking/coupling between the flapping wings and propellers’ propulsion modes when the propellers are behind the wings. It was also determined that the thrust generated by the propellers is not sensitive to the position of the assembly relative to the centerline of the platform. By adding propeller propulsion to Robo Raven, the augmented thrust increased the aerodynamic lift and the maximum weight of the vehicle, *mg*, as follows:

(4) $$\frac{C_{L}}{C_{D,p}}\left(k_{F}f^{2}S∆\alpha +C_{T}\rho \Omega_{}^{2}D^{4}-\Psi \right)=mg$$

*C_L_* Is the lift coefficient, *C_D_*_,*p*_ is the drag coefficient of the wing, *k_F_* is the flapping constant, *S* is the planform area of the wing, Δα is the flapping amplitude, *C_T_* is the thrust coefficient for the prop, ρ is the density of air, Ω is the angular speed of the propeller, *D* is the propeller diameter, and Ψ is the reduction in thrust force from the coupling between the propulsion modes. Results indicated that using propellers in conjunction with the wings can increase thrust as much as 261% at full signal. The resulting increase in thrust increases the available payload by a factor of over 5X, from 43.8 g to 272.9 g.

The propeller assembly did increase current draw by 176% when operating at 100% operational power, but the gain in available payload enabled more battery mass that increased the flight time by 341% over the initial design. The measured enhancements conformed to modeling predictions, while the effects of different mixed-mode operating conditions (e.g., flapping frequency and propeller speed) on flight time could also be ascertained. With mixed-mode propulsion, it was also possible to perform many maneuvers, (e.g., gliding, diving or loops) simply by adjusting the static position of each wing, which enabled us to investigate the effects of out-of-plane morphing on in-flight performance. Robo Raven V also became the first robot bird capable of taking off from the ground without assistance.

## 7. Conclusions and Future Directions

Our work on five generations of Robo Raven platform has demonstrated several new capabilities in the context of FWAVs. Collectively, different members of the Robo Raven family address requirements related to control autonomy, energy autonomy, and maneuverability. Our investigations have provided new insights and simplified models that have laid the foundation we hope inspires and challenges a new generation of researchers to explore this area further and create engineered platforms that can truly rival capabilities of biological creatures in terms of maneuverability, energy efficiency, and intelligence. By experimenting with flexible solar cells and propeller-assisted thrust generation, we have found that engineering of components that are compatible with purely biologically inspired concepts have the potential to significantly augment their capabilities. Investigations into the performance of highly compliant wings for flapping have enabled us to inform the FWAV community to take advantage of the versatility in flight performance afforded by adjustments to the wing kinematics in order to better accomplish mission objectives. Gaining a detailed understanding of how the motor, wings, and flapping gait will respond to changes in their respective operating conditions is a use framework to optimize the performance of FWAVs.

The Robo Raven family was able to achieve these advances in FWAV performance through the following contributions:

- Robo Raven I—utilized programmable servo motors to independently control the flapping profile for highly compliant wings in order to successfully perform a variety of complex maneuvers, including dives, flips, and buttonhook turns.
- Robo Raven II—through a system-level simulation focusing on actuator, battery, and wing subsystems, it was possible to achieve a more optimal flapping profile to transform electrical energy into aerodynamic force for greater endurance.
- Robo Raven III—was the first FWAV to utilize multifunctional wings to enhance endurance and to identify a potential pathway to energy autonomy through the harvesting of solar energy.
- Robo Raven IV—an on-board decision-making capability based on plate modeling of the aerodynamic performance of the platform was developed that enabled autonomous maneuvers, such as flying to a desired location and then performing a dive to interrogate it more closely.
- Robo Raven V—was the first FWAV that exploited the benefits of fixed and flapping wing flight through using propellers for mixed-mode propulsion to augment thrust, resulting in increased payload capacity and greater flight time, while also enhancing maneuverability at higher flight speeds than achieved through a pure flapping mode.

Table 3 below shows comparison among different versions of Robo Ravens.

While inspiration from birds was useful in helping to arrive at a flying prototype, there are still key differences between flying animals and Robo Raven that illuminate potential research areas needing additional work. Key areas for future research are summarized below:

- Wing Concepts: The wings used on Robo Raven are extremely thin relative to a bird’s and still have fewer degrees of freedom due to our choice of a single actuator per wing. Robo Raven’s wings also lack the fine control achieved by birds through the use of feathers, and the twist dynamics are a passive function of the forces imposed on them as opposed to a system with multiple degrees of freedom, as in birds. Future directions of research include increasing controllable degrees of freedom for wings to support a richer variety of flight modes and the use of feathers to control aerodynamics characteristics of wings [64]. New advances in actuator technology also need to be explored to achieve these capabilities. Utilizing much thicker wings while maintaining the compliance needed to generate greater aerodynamic force at low Reynolds number flows will require the use of advanced composite materials in wing design. Multifunctional materials and engineered components will need to be utilized so that wing structures can also be used for non-structural purposes.
- Bodies and Appendages: Most of the research in avian-inspired vehicles has focused on wing designs. Birds also use their bodies to control flight, particularly for maneuvers such as hovering. Research in aerodynamic and articulating body designs can further improve performance. Additional appendages up the bodies such as claws and legs can offer new capabilities to these flying vehicles, including multimodal locomotion. These capabilities would enable the FWAV to perform various tasks, such as landing, perching, walking, and self-powered take-offs. For instance, when the power is running low, the MAV could perch on a tree and recharge its battery using an onboard solar cell. Similarly, if the MAV is tracking a stationary target, it could land and conserve battery life until further movement is detected.
- Energy Storage: Enhancing the energy storage capacity of batteries or alternative power sources employed in compact FWAVs can enable smaller flight platforms to carry larger payloads. Presently, the limited flight duration poses a significant constraint on the widespread adoption of small FWAVs. Through ongoing exploration and development of advanced power storage technologies, the potential for FWAVs to serve a broader array of applications can be fully unlocked. Improving the energy density of batteries and refining electrical energy delivery via more elastic energy storage and release (i.e., reduced hysteresis) can lead to enhanced flight endurance for FWAVs. These advancements have the potential to create a whole new category of flapping fliers with practical and useful flight endurance.
- Additional Flight Modes: Larger FWAVs could adopt additional mechanisms to enhance their glide performance, such as exploiting thermals and soaring, much like larger birds do. This will require developing new sensors and control strategies that enable the platform to interrogate its environment and efficiently interact with it to reduce energy consumption, similar to real birds that are capable of migrating over thousands of miles.

## Figures and Tables

**Table 1:** Comparison of Robo Raven with Ravens.

Raven Specs		Robo Raven Specs	
Length:	24 to 26 in (61 to 66 cm)	Length:	24 in (61 cm)
Wingspan:	45.6 to 56.4 in (1.2 to 1.4 m)	Wingspan:	44 in (1.1 m)
Weight:	2.3 lbs (1.3 kg)	Weight (w/battery, w/out):	(291.6 g, 264.5 g)
Flapping frequency:	4–6 Hz	Flapping frequency:	4 Hz

**Table 2:** The more accurate kinematics predicted by the coupled model reduces errors in the prediction of lift, torque, and power consumption.

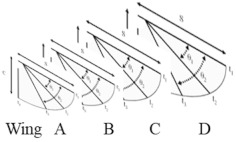												
	Flight Testing				Coupled Model				Kinematics Only			
	A	B	C	D	A	B	C	D	A	B	C	D
**Avg. Lift** **(N)**	2.59	3.15	3.47	3.14	2.50	3.08	3.19	3.31	2.51	4.03	4.75	6.42
**Avg. Thrust** **(N)**	1.72	2.06	2.19	2.32	1.16	1.15	1.37	2.19	1.16	2.88	3.95	7.15
**Avg. Power** **(W)**	13.6	15.1	15.9	17.6	12.9	18.3	20.0	22.1	13.0	29.8	39.9	70.9
**Avg. Torque** **(N-m)**	0.99	1.05	1.06	1.17	0.70	1.02	1.15	1.31	1.43	2.30	2.70	3.64
**Max. Plunge** **Velocity** **(rad/s)**	14.69	14.04	13.60	12.61	16.69	15.02	12.96	11.56	20	20	20	20

**Table 3:** Comparison of Robo Raven Platforms.

	I	II	III	IV and V
Mass w/o Battery (g)	285.0	301.6	317.0	438.1
Maximum Takeoff Mass (g)	328.8	382.0	388.0	711.0
Wingspan (m)	1.168	1.33	1.33	1.168
Flight Speed (m/s)	6.7	6.2	5.6	10
